# Performance, Intake, Feed Efficiency, and Carcass Characteristics of Young Nellore Heifers under Different Days on Feed in the Feedlot

**DOI:** 10.3390/ani13132238

**Published:** 2023-07-07

**Authors:** Igor Machado Ferreira, Karla Alves Oliveira, Iorrano Andrade Cidrini, Mateus José Inácio de Abreu, Luciana Melo Sousa, Luis Henrique Cursino Batista, Bruno Grossi Costa Homem, Laura Franco Prados, Gustavo Rezende Siqueira, Flávio Dutra de Resende

**Affiliations:** 1 Departament of Animal Science, Faculty of Agricultural and Veterinary Science, São Paulo State University (UNESP), Jaboticabal 14884-900, São Paulo, Brazil; 2 Embrapa Agrobiologia, Seropédica 23891-000, Rio de Janeiro, Brazil; 3 Agência Paulista de Tecnologia dos Agronegócios (APTA), Colina 14770-000, São Paulo, Brazil

**Keywords:** backfat thickness, carcass gain, days on feed, feed efficiency, Nellore heifers

## Abstract

**Simple Summary:**

Brazilian feedlot operators subjectively decide how long animals must stay in the feedlot based on a visual evaluation of weight and fatness. Typically, remuneration for commodity meat producers is based on carcass weight, and the lack of a metric to define the ideal slaughter endpoint often leads to erroneous decision-making, mainly due to not knowing the actual carcass gain of the animals. The use of “days on feed” (DOF) is a metric that allows the producer to precisely determine when the animal’s gain drops and, consequently, the feed efficiency in the feedlot. Thus, this study aimed to evaluate the effect of DOF on the performance, intake, feed efficiency, and carcass characteristics of Nellore heifers that received high supplementation during the growing phase. This study generated information that can help to determine the biological slaughter endpoint for young heifers finished in the feedlot. Our results suggest a relationship between the proportion of ether extract of the shrunk body weight, backfat thickness, and body weight can be used as a reference point to make decisions about the biological endpoint of slaughter in the feedlot based on days on feed.

**Abstract:**

Two studies evaluated the productive characteristics of young Nellore heifers receiving different days on feed (DOF) to determine the biological slaughter endpoint. In Experiment 1 (Exp. 1), fifty-one Nellore heifers [324 ± 19.3 kg of body weight (BW); 16 ± 1 months] were split into three DOF lengths (45, 75, or 105 days), while in Experiment 2 (Exp. 2), thirty-six Nellore heifers (362 ± 25.5 kg of BW; 18 ± 1 months of age) were split into three DOF lengths (45, 90, or 135 days). In both studies, all animals were distributed in complete randomized blocks according to initial BW and stratified via carcass ultrasound. The diet was supplied ad libitum, allowing 3% of refusals. The point at which the animals achieved 25% of ether extract of shrunk body weight (EE_SBW_) was defined as the biological endpoint. Thus, relationships were made between some characteristics obtained in both studies. Positive linear relationships were found between backfat thickness (BFT) vs. EE_SBW_ (*p* < 0.001, *r* = 0.84) and BFT vs. body weight (*p* < 0.001, *r* = 0.77). Our results suggest that the biological slaughter endpoint for young Nellore heifers is 6.97 mm of backfat thickness or 402 kg shrunk body weight, corresponding to around 75 DOF.

## 1. Introduction

In recent years, an increase in the demand for meat has been observed worldwide [1]. Brazil represents a crucial player in this context, where according to USDA projections [2], meat production in terms of tonnes of produced carcass equivalent is expected to increase by 9% in Brazil until 2028 (representing 23% of total world exports). It is known that Brazilian beef production is characterized by animals finished on pasture receiving only mineral salt [3]. As a result, the age at slaughter and the age at first calving are higher compared to more intensive production systems (e.g., United States). Therefore, to meet growing demand, significant changes need to be adopted in the Brazilian production system, where the producer needs to adopt intensive systems to obtain heavy animals in a short time, reducing the age at slaughter. In the upcoming years, a tech-enhanced, intensive, short-cycle beef production chain should be the expected reality, according to Malafaia et al. [4].

To obtain pregnant heifers at 14 months of age, nutritional strategies with greater use of concentrated feed in the growth phase have been proposed as a means to shorten the age at first calving in Brazilian beef cattle production systems [5,6,7]. However, the pregnancy rate in these systems is partial, and heifers that do not get pregnant in the breeding season (open heifers) become cull heifers due to reproductive failure and can be used for meat production. These animals have favorable characteristics (weight and body condition) to be finished at the end of the growing season. In this sense, heifers slaughtered at less than 24 months of age increased by 6.56% over the last 23 years (from 1997 to 2020), accounting for 10.6% of the total number of animals slaughtered annually in Brazil in 2020 [8].

The influence of days on feed (DOF) on heifers’ performance and carcass characteristics was initially reported in the literature for *Bos taurus taurus* animals in the 1970s [9]. These studies provide a better understanding of the dynamics of tissue deposition [10,11], making it possible to obtain feed conversion efficiency into carcass throughout the finishing process. However, DOF’s effect on animal performance characteristics and the carcass tissue deposition of young Nellore heifers is still limited. Thus, there has to be more information in the Brazilian literature about changes in carcass deposition during the feedlot stage, mainly when animals receive a significant amount of supplement during the background phase. As a result, producers are sending animals to slaughter earlier and lighter. Furthermore, a lack of understanding about the carcass deposition process in feedlot can make the activity unfeasible, due to the non-use of a higher final weight and perhaps the receipt of higher values resulting from commercialization in specific meat quality markets [11]. According to Andrade et al. [12], no studies report characteristics that help the producer decide how many days animals need to stay in the feedlot. Most of the time, Brazilian producers make the decision subjectively.

Thus, this study aimed to assess DOF’s effect on performance, intake, feed efficiency, and carcass characteristics of Nellore heifers that received high supplementation during the growing phase, generating information that can be used to determine the biological slaughter endpoint for young Nellore heifers finished in feedlots. We hypothesized that the increase in DOF in the feedlot would reduce weight gain and feed efficiency due to an increase in carcass fat deposition. In addition, the relationship between the fat proportion in the empty body weight and subcutaneous fat thickness are characteristics that would help determine the slaughter biological point.

## 2. Materials and Methods

### 2.1. Location

Two experiments were conducted in the experimental feedlot at Agência Paulista de Tecnologia dos Agronegócios (APTA), Alta Mogiana Regional pole, Colina, São Paulo, Brazil (geographic coordinates: 20°42′50.0″ S 48°32′52.7″ W). The experiments were approved by the Ethics Committee on the Use of Animals (Protocols nº 0003/2020 and 0004/2020). Both experiments were carried out during the dry season. Experiment 1 was carried out from June 2020 to September 2020 (totaling 105 days), and Experiment 2 from April 2021 to August 2021 (totaling 135 days). All heifers used in both studies were reared on the same pasture conditions for eight months and received energy protein supplements (72% of total digestible nutrients and 24.7% of crude protein) at a rate of 1% of body weight (BW; average 2.22 kg supplement/heifers/d, as feed basis). The heifers had an average daily gain (ADG) of 0.679 kg during this period.

### 2.2. Experiment 1

Three different feedlot times were evaluated (DOF: 45, 75, and 105 days). Fifty-one Nellore heifers [324 ± 19.3 kg of BW; 16 ± 1 months of age], blocked according to initial BW and stratified via carcass ultrasound, were assigned randomly within the block to pens; each pen was randomly assigned to one of the three treatments. Before housing the animals, they were vaccinated and dewormed (Biopoligen^®^ HS, Policlostrigen^®^ e Biopersol Forte M. V., Biogénesis Bagó, Curitiba, Paraná, Brazil). Subsequently, the animals were placed into 18 pens measuring 6 × 15 m [17 heifers/treatment; six pens/treatment (five pens with three heifers and one pen with two heifers)] in an open-air feedlot with shared water fountains (500 L capacity) between two pens equipped with a high flow valve. The pens were considered experimental units. At the end of each feedlot period, heifers were weighed to obtain the shrunk final BW (after 16 h of feed and water withdrawal), and ultrasound measurements were taken on the carcass. On the following day, the heifers were loaded onto trucks and transported to a commercial slaughterhouse (Minerva Foods, Barretos, SP, Brazil) located 34 km from the research facility. Upon arrival at the slaughterhouse, animals were kept in resting pens for 18 h (with free access to water) and then submitted to humanitarian slaughter according to the Brazilian RIISPOA (Regulation of Industrial and Sanitary of Animal Products), following the standard practices of Brazilian Federal Inspection. The hot carcass weight (HCW) was recorded.

### 2.3. Experiment 2

Thirty-six Nellore heifers (362 ± 25.5 kg of BW; 16 ± 1 months of age), blocked according to initial BW and stratified via carcass ultrasound were assigned randomly within the block to pens, and each pen was randomly assigned to one of the three different DOF (45, 90 and 135 days). The same procedure of the vaccination program carried out in Exp. 1 was adopted in Exp. 2. After this procedure, the animals were placed into 12 pens measuring 6 × 15 m [12 heifers/treatment; four pens/treatment and three heifers/pens) in an open-air feedlot with shared water fountains (500 L capacity) between two pens equipped with a high flow valve. Heifers were weighed at the end of each feedlot period to obtain the shrunk final BW, and ultrasound measurements were taken on the carcass. On the following day, the animals were slaughtered in the same commercial slaughterhouse as Exp. 1.

### 2.4. Diets and Feeding Management

The same diet was used in both experiments. The diet was formulated following nutritional requirements determined by the Cornell Net Carbohydrate and Protein System [13], for an estimated gain of 1.2 kg BW/d. An adaptation diet was provided to the animals during the first 15 days. On the first day of the adaptation period, 1% of BW was offered of diet, increasing 0.25% of the BW daily until the volume of feed refusals (orts) did not exceed 10% of what was offered every three days. When feed refusals exceeded 10%, the diet provided to the animals on the next day was decreased by 5%. Between the 16th and 22nd day, a transition diet was offered. This procedure was adopted to avoid an abrupt decrease in feed intake. From the 23rd to the last day, a finishing diet was provided to animals in both experiments. In all diets, the main roughage used was whole-plant corn silage. Furthermore, it is important to emphasize that there were no adjustments in the diet formulation during the study. No adjustments in the diet formulation were made to avoid favoring the treatments with a longer DOF, which could confound the treatments with a shorter DOF due to differences in energy intake. The animals in both experiments were fed daily at 8:30 am. A mixer wagon (RX-40 E; Casale^®^, São Carlos, SP, Brazil: capacity of 6.5 m^3^) equipped with a scale was used to distribute the diets in the pens.

Samples of individual ingredients were collected every three days throughout the experimental period. At the end of the experiment, all the samples were mixed, and a composite sample was created for each ingredient, taking into account the duration of each diet’s supply period (Table 1). These samples were dried in a forced-air circulation oven set at 55 °C for 72 h, ground through a Wiley mill (Thomas Model 4 Wiley, Thomas Scientific, Swedesboro, NJ, USA) to pass through 2 and 1 mm sieves, and then stored for chemical analysis. The dry matter (method 934.01), ash (method 942.05), crude protein (CP, method 978.04), and ether extract (EE, method 920.39) contents were measured according to the AOAC [14]. Organic matter (OM) was calculated as 100-ash. The neutral detergent fiber (NDF) and acid detergent fiber (ADF) contents were determined via sequential analysis as described by Van Soest et al. [15], using a fiber analyzer (TE-149, Tecnal, Piracicaba, SP, Brazil). Total digestible nutrient (TDN) values were estimated using the individual composition of feedstuff, according to Valadares Filho et al. [16].

### 2.5. Experimental Evaluation

#### 2.5.1. Intake and Animal Performance

Feedstuffs and refusals were weighed daily and collected thrice weekly for dry matter analysis and adjustment of each pen’s intake. The individual dry matter intake (DMI) was calculated from daily diets and subtracted from the refusals of the next day, divided by the number of animals/pens throughout the experimental period. The heifer BW was recorded at the beginning and end of 45, 75, 90, 105, and 135 DOF using a digital scale (model RUDD 300, Coimma LTDA, Dracena, SP, Brazil). The animals’ ADG was calculated as follows:

ADG, kg/d = (final BW − initial BW)/feedlot days, (1)

The feed efficiency (FE) was calculated as the ratio of ADG and DMI.

#### 2.5.2. Carcass Ultrassond

Carcass ultrasound was performed on the same day that animal performance was measured. A veterinary ultrasound Piemedical—Scanner 200 with a linear probe (ASP-18) and frequency of 3.5 MHz was used. The longissimus muscle area (LMA; cm^2^), backfat thickness (BFT, mm), and marbling (points 1 to 10) were measured through ultrasound images. Images were taken between the 12th and 13th ribs, transverse to the Longissimus dorsi muscle. Thus, the fat thickness was measured in the distal middle third of the rib eye area. Rump fat thickness (RFT) was measured in the longitudinal position between the ileum and ischium bones in the rump (junction of the gluteus medius and biceps femoris muscles). Vegetable oil was used as an acoustic coupling in all evaluations.

#### 2.5.3. Slaughter and Carcass Characteristics

At the beginning of Exp. 2, six heifers (two light heifers, two medium-weight heifers, and two heavy heifers) were slaughtered and used as a reference for the initial BW (mean = 360 ± 26.6 kg) and HCW (mean = 195 ± 17.3 kg). This procedure was adopted to compare the animals on the same basis. A regression equation for the shrunk BW and HCW of these heifers was used. The initial carcass weight (initial HCW) of the heifers was estimated as follows (Exp. 1: n = 51 and Exp. 2: n = 36):

y = 0.6362 (± 0.064)x − 34.0467 (± 23.13); R^2^ = 0.961; RMSE = 3.815, (2)

where y is the initial carcass weight (kg) and x is the shrunk initial BW (kg).

At the end of the feedlot period, each treatment’s heifers were slaughtered as previously described. After the slaughter, all carcasses were individually identified and weighed for HCW, and the dressing percent was calculated (DP, % = HCW/final BW). The carcass ADG was calculated as the difference between the final and initial HCW, divided by the number of feedlot days, according to the treatment. The carcass FE was calculated as the ratio between the carcass gain (CG) and the DMI. The carcass transfer (CT) was calculated using the equation proposed by Sampaio et al. [18] as follows:

CT, % = [(final HCW − initial HCW)/(final BW − initial BW)], (3)

The total carcass gain (TCG) was calculated using the equation:

TCG, kg = final HCW − initial HCW, (4)

After 24 h post-slaughter with cold-chamber storage (2 °C), all carcasses were weighed, and the cold carcass weight (CCW, kg) was measured. To quantify primal cuts of the carcass, the right half-carcass was separated into the forequarter (between the fifth and sixth rib), thin flank, and hindquarter, according to the Brazilian Beef Cuts Standards.

#### 2.5.4. Body Composition

The ether extract of the empty body weight (EE_EBW_, kg) was estimated using the exponential equation proposed by Marcondes et al. [19] for Nellore heifers:

EE_EBW_, kg = 1.959 × e^0.010 × EBW^, (5)

The proportion of EE_EBW_ was divided by SBW to quantify the proportion of EE_SBW_ (kg). Furthermore, the retained energy (RE) in the body was obtained according to the equation proposed by Valadares Filho et al. [16]:

ER, Mcal/d = 0.061 × EQEBW^0.75^ × EBG^1.035^, (6)

where EQEBW is the equivalent empty body weight. The empty body weight (EBW) was obtained through multiplying the shrunk body weight (SBW) by 0.891. The empty body weight gain (EBG) was estimated through multiplying 0.9630 × ADG^1.0115^, and the EQEBW was calculated according to the equation proposed by Valadares Filho et al. [16], considering the mature body weight for heifers of 402 kg. The RE percentage deposited as protein (%REp) was calculated using the equation proposed by Chizzotti et al. [20]:

REp, % = 10.1 + 166.7 × e^(−0.660×RE)^, (7)

### 2.6. Statistical Analysis

Animal performance, intake, body composition, primal cuts, carcass characteristics, and ultrasound measurement were analyzed in a randomized complete block design with DOF as fixed effects and pen and block as random effect. The pen was considered the experimental unit. Data were analyzed through fitting mixed models [21] using the MIXED procedure of SAS (SAS Institute, Cary, NC). Linear and quadratic polynomial contrasts were built to evaluate the DOF effects. Polynomial contrast coefficients were adjusted for unequally spaced treatments using the IML procedure of SAS (version 9.3; SAS Inst. Inc., Cary, NC). All data were considered normally distributed (Shapiro–Wilk test, W ≥ 0.80) using the UNIVARIATE procedure of SAS (SAS Inst., Inc., Cary, NC, USA). Degrees of freedom were estimated using the Kenward–Roger approach. Results were considered significant at *p* < 0.05.

Mean data from each experimental unit of the final HCW, BFT, carcass FE, marbling point and proportion of EE_SBW_ from each study were pooled and analyzed. The relationships between the final HCW, BFT, carcass FE, and marbling point (FE) with final BW and proportion of EE_SBW_ were determined using a regression analysis at 10% probability using the REG procedure of SAS (SAS Inst., Inc., Cary, NC, USA). Furthermore, Pearson correlation coefficients were calculated using the CORR procedure.

## 3. Results

Neither experiment found differences between the DOF for initial BW and HCW (*p* > 0.05; Table 2). The final BW and final HCW increased linearly over DOF (*p* < 0.01) in both studies. In Exp. 1, the ADG, FE, carcass ADG, and carcass FE were not influenced by treatments (*p* > 0.05; an average of 1.19 kg/d, 0.158 kg/kg, 0.799 kg/d, and 0.106, respectively). However, the DMI increased linearly over DOF (*p* = 0.001). On the other hand, there was a linear reduction for the ADG (*p* = 0.017), FE (*p* = 0.029), carcass ADG (*p* = 0.027), and carcass FE (*p* = 0.002) for Exp. 2. No differences between DOF were recorded for DMI in Exp. 2 (*p* > 0.05, an average of 8.3 kg of DM/d). Using more time in the feedlot increased the TCG and DP linearly in both experiments (*p* < 0.01). Regarding the effect of the DOF over CT, no differences were recorded in both experiments (*p* > 0.05, an average of 67.8% in Exp. 1 and 65.0% in Exp. 2).

For the carcass ultrasound measurements, the LMA, BFT, and RFT in both experiments and the marbling point in Exp. 2 increased linearly over the DOF (*p* < 0.05). Furthermore, the EE_EBW_ composition and RE (mcal/kg) increased linearly over the DOF (*p* < 0.001), while the protein proportion in the gain decreased linearly in both studies (*p* < 0.001).

Concerning the primal cuts, CCW values increased linearly over the DOF (*p* < 0.01, Table 3) in both experiments. In Exp. 1, a quadratic effect between DOF and the proportion of forequarter and hindquarter was reported (*p* = 0.011 and *p* < 0.001, respectively). The thin flank proportion increased linearly for Exp. 1 (*p* = 0.03). On the other hand, there was a linear increase in the thin flank proportion in Exp. 2. No differences were obtained between treatments in the forequarter and hindquarter proportions (*p* > 0.05; an average of 38.6% and 49%, respectively) in Exp. 2.

**Figure 1:**
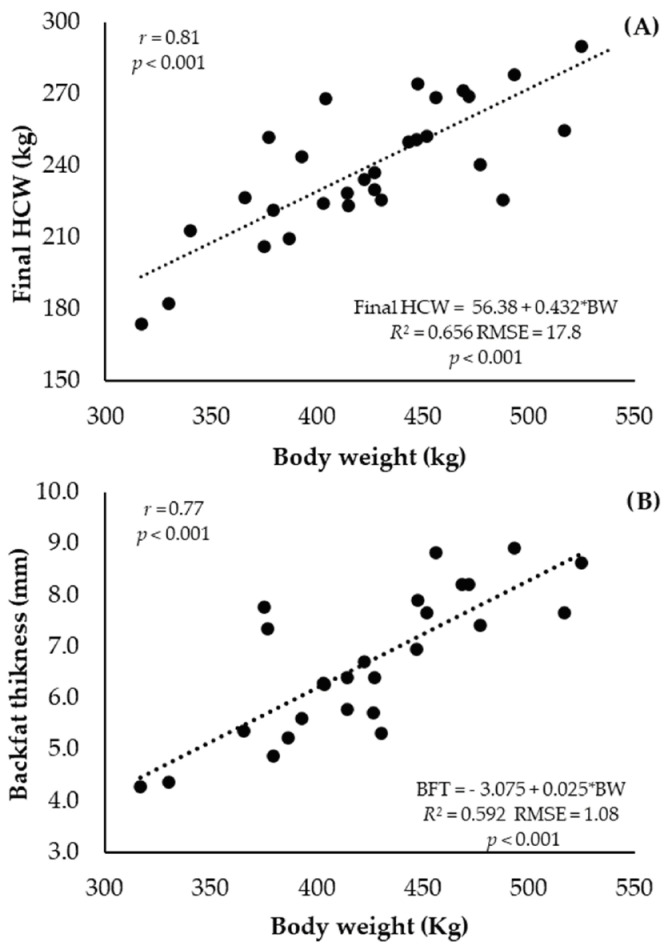
Final hot carcass weight ((**A**); HCW, kg) and backfat thickness ((**B**); BFT, mm) as a function of the body weight (BW, kg) of Nellore heifers that received high supplementation during the growing phase slaughtered at different feedlot days.

The final HCW (Figure 1A) and BFT (Figure 1B) varied according to the heifers’ BW (*p* < 0.001); a positive linear relationship was obtained between these variables with a strong correlation coefficient (*p* < 0.001, *r* = 0.81 and *r* = 0.77, respectively).

**Figure 2:**
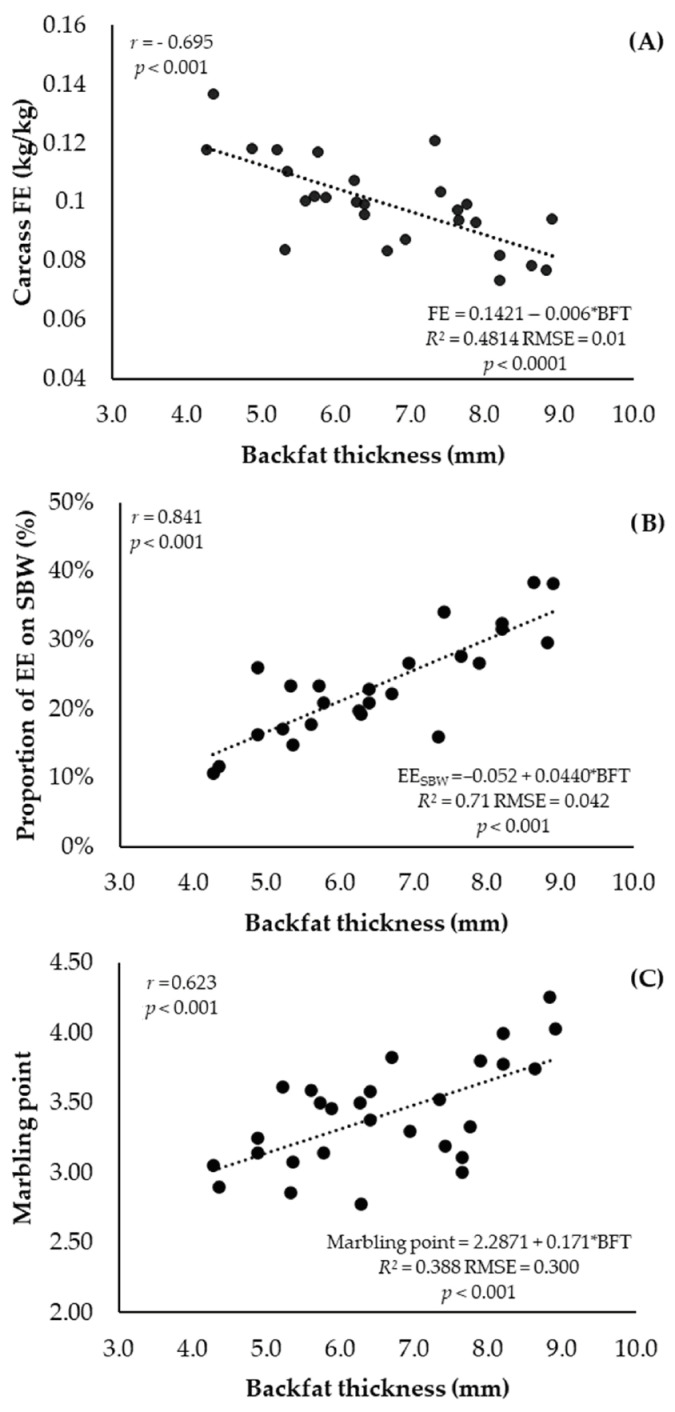
Carcass feed efficiency ((**A**); G:F, kg/kg), proportion of ether extract on the shrunk body weight ((**B**); SBW, kg), and marbling point ((**C**) as a function of the backfat thickness (BFT, mm) of Nellore heifers that received high supplementation during the growing phase slaughtered at different feedlot days.

The carcass FE varied according to BFT (Figure 2A), but a negative linear relationship was obtained (*p* < 0.001; *r* = −0.70). Furthermore, a positive linear relationship was obtained between EE_SBW_ (Figure 2B) and marbling point (Figure 2C) with BFT (*p* < 0.001, *r* = 0.84 and *r* = 0.623, respectively).

## 4. Discussion

Two consecutive studies were performed to evaluate DOF’s effects on the performance, intake, feed efficiency, and carcass characteristic of young Nellore heifers. Some Brazilian research strives to establish the optimal biological slaughter endpoint in feedlots for bulls (based on constant final body weight; [22,23]) and cull heifers older than 24 months of age [24]. However, there is no information for young Nellore heifers (16–18 months of age) who receive high supplementation (1% of BW) during the growing phase.

According to Silvestre and Millen [25], the typical feeding period used in Brazilian feedlots for heifers is 89 days. Based on this information, we determined our treatments. Therefore, we chose three different DOF lengths, since our initial hypothesis was that heifers in Exp. 1 would reduce performance and feed efficiency before 105 days. However, our hypothesis was not confirmed, and we decided to carry out Exp. 2, extending the feeding period. Although we did not achieve a group of animals with the same average initial weight between experiments, the main characteristics of Exp. 1 related to the heifers’ background phase were maintained.

It is important to highlight that there is a consensus in the literature about the negative effect of supplementation during the growing phase on the next phase’s performance [18,26,27,28,29]. According to Lancaster et al. [30], ADG during finishing is negatively correlated with background ADG. On the other hand, supplementation during the growing phase provides that the animals arrive heavier and fatter at the beginning of the feedlot phase, reducing the time required for the slaughter compared to animals that suffered feed restriction [31]. The slaughter of young animals (less than 18 months old) is a viable option for many producers due to increased market demand and higher carcass value from these animals when superior meat quality can be attributed to them [32].

The reduction in feed efficiency with the increase in DOF is associated with a greater energy requirement for gain [33,34], an increase in the energy requirement for maintenance, and a higher energetic cost for adipose tissue deposition [9]. Thus, a possible explanation for the absence of a reduction in the feed efficiency during 105 DOF in Exp. 1 is that the animals maintained their body and carcass weight gain. According to Silva et al. [35], animals that received a high level of supplementation during the growing phase have more metabolically active organs. As a result, effective nutrient utilization occurs in the skeletal muscle rather than the viscera during the feedlot period. Moreover, despite a reduction in the gain protein proportion, the substantial increase in DMI may have contributed to maintaining the gain and FE. The same cannot be assumed for Exp. 2, where an increase in DMI was not observed, and a possible explanation for the lack of effect on DMI in Exp. 2 is that the initial BW was greater in this experiment. Heavier heifers at the beginning of the feedlot negatively affect performance during the finishing phase [36]. However, these initial body weight values fall within the weight range commonly practiced in heifer feedlots in Brazil [25,37].

Brazilian feedlot operators subjectively determine the slaughter endpoint based on a visual assessment of weight and fat content, yet 85% of feeders recognized that their current method of slaughter endpoint identification needs to be improved [12]. Based on this, we used the relationship between carcass variables measured throughout the trial to determine the best time for the biological slaughter endpoint for heifers finished in the feedlot. A positive linear correlation was recorded between the proportion of EE_SBW_ and BFT. Thus, when the heifers obtained 25% of EE in the SBW [38], the animals presented 6.97 mm of BFT. Subsequently, the correlation between BFT and BW indicated that when animals obtained 6.97 mm of BFT, they showed 402 kg of BW and 230 kg of carcass weight. These points are important and agree with the maturity weight defined by Valadares Filho et al. [16] for Nellore females. Thus, we advise against allowing young heifers who have received high supplementation during the growing phase to exceed 75 days on feed (DOF), 6.97 mm backfat thickness (BFT), or 402 kg body weight (BW) as a biological endpoint for slaughter for the commodity market. These recommendations align with the criteria of the majority of slaughterhouses in Brazil, which typically require a body weight of 180 to 230 kg and a minimum backfat thickness of 3 mm.

On the other hand, in a production system with the objective of increasing meat quality and premium carcass, heifers fed in feedlot for more than 75 DOF may be an option. The linear positive relationship between BFT and marbling point can confirm this. However, the coefficient correlation obtained in the current experiments is lower than the value reported by May et al. [39], who found a value of 0.81 for the correlation between the marbling point and BFT of crossbred Angus × Hereford. In the same sense, Pflanzer and Felício [40] observed that backfat thickness affected the amount of visible intramuscular fat (marbling) in steaks from Nellore steers, where animals with 4 to 6 mm of BFT showed a higher value of marbling than animals with 1 to 3 mm. Another important point is that animals typically finished in Brazilian feedlots receive almost no starch supplementation during the growing phase and are slaughtered below mature body weight [15]. This is the reason why there is little discussion about the marbling increase and benefits in the meat quality of *Bos taurus indicus* animals in Brazil. Subcutaneous deposits are known to be stored and formed before intramuscular fat deposits, known as marbling [41]. Therefore, the heifers’ dietary history, as well as the high value of subcutaneous fat at the beginning of feedlot may have contributed to the differentiation of intramuscular fat deposits in young Nellore heifers.

The heifers increased the thin flank proportion linearly over DOF. Similar results were found by Rezende et al. [24] evaluating the carcass characteristics of Nellore heifers slaughtered at different finishing weights in the feedlot. The increase in thin flank proportion may be due to the greater capacity of females for fat deposition in the ribs [10,23,42]. The values of the primary cuts in the current study are within the range considered ideal postulated by Luchiari Filho et al. [43], in which the proportion of the hindquarters must be greater than 48%, the forequarters up to 39%, and the thin flank up to 13%. Although there was a significant increase in the hindquarter proportion in Exp. 1, the proportion was above 48% in both studies. Increasing the hindquarter proportion is advantageous due to the location of prime cuts with greater commercial value [44]. Furthermore, it is important to highlight the lack of studies evaluating deboning yield for heifers receiving different DOF in the feedlot. Although an increase in carcass weight benefits the industry [45], excessively increasing carcass fat coverage can increase the filleting cost of commercial cuts.

## 5. Conclusions

Backfat thickness and body weight can be used as a reference point to decide the biological slaughter endpoint of young Nellore heifers in feedlot. Our results suggest that the biological slaughter endpoint for young Nellore heifers is 6.97 mm of backfat thickness or 402 kg of shrunk body weight, corresponding to around 75 DOF. Additional studies should focus on the cost effectiveness of production systems that use longer feedlot periods to improve meat quality and deboning yield of young Nellore heifers for the industry.

## Figures and Tables

**Table 1:** Diet composition offered in both experiments.

Ingredient	Diets		
	Adaptation ^1^	Transition ^2^	Finishing ^3^
Ingredient, %DM			
Whole plant corn silage	56.5	40.5	24.6
Soybean meal	10.0	7.00	4.00
Ground corn	29.9	47.2	64.4
Calcium salts ^4^	1.00	2.25	3.50
Mineral supplement ^5^	2.60	3.05	3.50
Nutritional profile, %DM			
Dry matter	52.4	57.8	63.4
Organic matter	93.9	94.6	95.2
Crude Protein	16.0	15.6	14.5
Neutral detergent fiber	33.1	27.6	21.2
Acid detergent fiber	17.5	14.4	10.0
Ether extract	3.32	4.39	4.47
Ash	6.11	5.37	4.81
Total digestible nutrients ^6^	79.0	82.5	86.0
ME, mcal/kg ^7^	2.85	2.98	3.10

^1^ Diet provided to animals in the first 15 days. ^2^ Diet provided to animals from the 15th to the 22nd day. ^3^ Diet provided to animals from the 23rd to the 105th day in Exp. 1 and the 135th day in Exp. 2. ^4^ Rumen-protected fat, calcium salts of soybean fatty acids (Vaccinar, Belo Horizonte, MG, Brazil). ^5^ Assurance levels per kilogram of products: Ca: 90 g, P: 10 g, Na: 40 g, S: 17 g, Zn: 1060 mg, Cu: 285 mg, Fl: 167 mg, Mg: 825 mg, Co: 17 mg, I: 21 mg, Se: 5 mg, sodium monensin: 630 mg, Urea: 355.9 g (Trow Nutrition, Campinas, SP, Brazil). ^6^ Total digestible nutrients (TDN) was estimated using the individual feedstuff composition according to Valadares Filho et al. [16]. ^7^ ME = TDN (g/kg DM) × 4.4 × 0.82 [17].

**Table 2:** Performance, intake, carcass gain, and feed conversion rate of Nellore heifers that received high supplementation during the growing phase and slaughtered at different feedlot days.

Item	Exp. 1						Exp. 2					
	DOF				Effect		DOF				Effect	
	45	75	105	SEM	L	Q	45	90	135	SEM	L	Q
Performance: live basis												
Initial BW, kg	325	325	321	19.3	0.102	0.288	361	359	362	14.2	0.328	0.075
Final BW, kg	374	411	442	20.3	<0.001	0.432	414	462	504	16.7	<0.001	0.689
ADG, kg/d	1.17	1.21	1.20	0.046	0.652	0.644	1.25	1.19	1.03	0.055	0.017	0.503
DMI, Kg/d	7.1	7.68	7.79	0.212	0.001	0.099	8.20	8.47	8.23	0.300	0.921	0.427
FE, kg/kg	0.164	0.157	0.154	0.006	0.193	0.746	0.148	0.139	0.125	0.006	0.029	0.748
Performance: Carcass basis												
Initial HCW, kg	173	173	170	5.05	0.991	0.886	195	194	196	9.05	0.612	0.411
Final HCW, kg	207	229	251	11.7	<0.001	0.830	229	259	283	9.44	<0.001	0.564
Carcass gain, kg/d	0.805	0.792	0.801	0.032	0.232	0.812	0.758	0.716	0.650	0.039	0.027	0.750
TCG, kg	33.8	56.2	80.9	1.79	<0.001	0.853	33.9	64.5	87.8	3.56	<0.001	0.428
Carcass FE, kg/kg	0.114	0.103	0.103	0.005	0.086	0.283	0.092	0.084	0.078	0.003	0.002	0.293
CT ^1^, %	69.5	66,7	67.2	1.94	0.405	0.275	66.2	63.9	65.0	2.17	0.719	0.535
DP, %	55.2	55.7	56.8	0.003	0.004	0.434	55.4	56.2	57.1	0.412	0.006	0.895
Carcass Ultrasound measurements												
LMA, cm	64.3	68.5	73.3	2.80	<0.001	0.852	65.4	82.5	85.5	1.76	<0.001	0.010
BFT, mm	5.05	5.69	7.04	0.400	<0.001	0.318	6.33	7.69	9.77	0.620	0.036	0.647
Marbling, points	3.11	3.47	3.44	0.134	0.078	0.210	3.23	3.78	4.03	0.160	0.007	0.463
RFT, mm	8.01	10.0	10.8	0.711	<0.001	0.284	10.3	12.3	13.3	0.710	0.011	0.533
Body composition												
EE_EBW_^2^, %	16.4	22.0	27.0	3.34	<0.001	0.817	21.0	29.7	35.4	2.83	<0.001	0.481
RE, mcal/kg	5.57	6.03	6.39	0.237	<0.001	0.620	6.10	6.73	7.31	0.182	<0.001	0.695
% REp	14.6	13.4	12.8	0.527	<0.001	0.269	13.2	12.1	11.5	0.252	<0.001	0.04

L = linear effect; Q = quadratic effect; SEM = standard error of the means; BW = body weight after 16 h of feed and water withdrawal; ADG = average daily gain; DMI = dry matter intake; TCG = total carcass gain; G:F = feed efficiency; DP = dressing percentage; LMA = longissimus muscle area; BFT = backfat thickness, RFT = rump fat thickness. ^1^ Carcass transfer = [(final HCW − initial HCW)/(final BW − initial BW)] × 100 [18]; ^2^ EE_EBW_, % = 1.959 × e^0.010 × EBW^ [19], where EBW = empty body weight; RE (Mcal/kg) = (0.061*EQEBW^0.75^ × EBG^1.035^) [16], where RE = energy retain, EQEBW = equivalent empty body weight and EBG = empty body gain; % REp = 10.1 + 166.7 × e(^−0.660×RE^) [20].

**Table 3:** Primal cuts from carcasses of Nellore heifers that received high supplementation during the growing phase slaughtered at different feedlot days.

Item	Exp. 1						Exp. 2					
	DOF				Effect		DOF				Effect	
	45	75	105	SEM	L	Q	45	90	135	SEM	L	Q
CCW, kg	206	228	244	11.8	<0.001	0.830	225	255	278	9.38	<0.001	0.422
Forequarter, %	40.5	36.9	37.6	0.475	<0.001	0.011	38.6	39.1	38.1	0.290	0.238	0.071
Thin flank, %	11.4	12.7	12.5	0.320	0.030	0.069	12.1	12.8	13.8	0.300	0.002	0.663
Hindquarter, %	48.1	50.4	49.9	0.344	<0.001	<0.001	49.4	48.2	49.5	0.690	0.893	0.147

SEM = standard error of the mean; L = linear effect; Q = quadratic effects; CCW = cold carcass weight.

## Data Availability

Data available on request due to privacy or ethical restrictions.

## References

[1] McAlpine C.A., Etter A., Fearnside P.M., Seabrook L., Laurance W.F. (2009). Increasing World Consumption of Beef as a Driver of Regional and Global Change: A Call for Policy Action Based on Evidence from Queensland (Australia), Colombia and Brazil. Glob. Environ. Chang..

[2] USDA Agricultural Projection to 2028. https://www.ers.usda.gov/webdocs/outlooks/92600/oce-2019-1.pdf?v=9842.3.

[3] Millen D.D., Pacheco R.D.L., Meyer P.M., Rodrigues P.H.M., De Beni Arrigoni M. (2011). Current Outlook and Future Perspectives of Beef Production in Brazil. Anim. Front..

[4] Malafaia G.C., Mores G.d.V., Casagranda Y.G., Barcellos J.O.J., Costa F.P. (2021). The Brazilian Beef Cattle Supply Chain in the next Decades. Livest. Sci..

[5] Cappellozza B.I., Cooke R.F., Guarnieri Filho T.A., Bohnert D.W. (2014). Supplementation Based on Protein or Energy Ingredients to Beef Cattle Consuming Low-Quality Cool-Season Forages: I. Forage Disappearance Parameters in Rumen-Fistulaed Steers and Physiological Responses in Pregnant Heifers. J. Anim. Sci..

[6] Moriel P., Lancaster P., Lamb G.C., Vendramini J.M.B., Arthington J.D. (2017). Effects of Post-Weaning Growth Rate and Puberty Induction Protocol on Reproductive Performance of -Influenced Beef Heifers. J. Anim. Sci..

[7] Moriel P., Cooke R.F., Bohnert D.W., Vendramini J.M.B., Arthington J.D. (2012). Effects of Energy Supplementation Frequency and Forage Quality on Performance, Reproductive, and Physiological Responses of Replacement Beef Heifers. J. Anim. Sci..

[8] Instituto Brasileiro de Geografia e Estatística—Sistema de Recuraperação Automática (SIDRA). https://sidra.ibge.gov.br/tabela/1092#resultado.

[9] Zinn D., Durham R.M., Hedrick H.B. (1970). Feedlot and Carcass Grade Characteristics of Steers and Heifers as Influenced by Days on Feed. J. Anim. Sci..

[10] Owens F.N., Gill D.R., Secrist D.S., Coleman S.W. (1995). Review of Some Aspects of Growth and Development of Feedlot Cattle. J. Anim. Sci..

[11] Owens F.N., Dubeski P., Hanson C.F. (1993). Factors That Alter the Growth and Development of Ruminants. J. Anim. Sci..

[12] De Andrade T.S., Albertini T.Z., Barioni L.G., Medeiros S.R., Millen D.D., dos Santos A.C.R., Goulart R.S., Lanna D.P.D. (2020). Perception of Consultants, Feedlot Owners, and Packers Regarding the Optimal Economic Slaughter Endpoint in Feedlots (Part I): A National Survey in Brazil. Can. J. Anim. Sci..

[13] Fox D.G., Tedeschi L.O., Tylutki T.P., Russell J.B., Van Amburgh M.E., Chase L.E., Pell A.N., Overton T.R. (2004). The Cornell Net Carbohydrate and Protein System Model for Evaluating Herd Nutrition and Nutrient Excretion. Anim. Feed Sci. Technol..

[14] AOAC (2000). Official Methods of Analysis of the Association of Analytical Chemists International.

[15] Van Soest P.J., Robertson J.B., Lewis B.A. (1991). Methods for Dietary Fiber, Neutral Detergent Fiber, and Nonstarch Polysaccharides in Relation to Animal Nutrition. J. Dairy Sci..

[16] Valadares Filho S.C., Costa e Silva L.F., Gionbelli M.P., Rotta P.P., Marcondes M.I., Chizzotti M.L., Prados L.F. (2016). Exigências Nutricionais de Zebuínos Puros e Cruzados.

[17] Carvalho J.R.R., Chizzotti M.L., Schoonmaker J.P., Teixeira P.D., Lopes R.C., Oliveira C.V.R., Ladeira M.M. (2016). Performance, Carcass Characteristics, and Ruminal PH of Nellore and Angus Young Bulls Fed a Whole Shelled Corn Diet. J. Anim. Sci..

[18] Sampaio R.L., de Resende F.D., Reis R.A., de Oliveira I.M., Custódio L., Fernandes R.M., Pazdiora R.D., Siqueira G.R. (2017). The Nutritional Interrelationship between the Growing and Finishing Phases in Crossbred Cattle Raised in a Tropical System. Trop. Anim. Health Prod..

[19] Marcondes M.I., Tedeschi L.O., Valadares Filho S.d.C., Costa e Silva L.F., Silva A.L. (2016). Using Growth and Body Composition to Determine Weight at Maturity in Nellore Cattle. Anim. Prod. Sci..

[20] Chizzotti M.L., Tedeschi L.O., Valadares Filho S.C. (2008). A Meta-Analysis of Energy and Protein Requirements for Maintenance and Growth of Nellore Cattle. J. Anim. Sci..

[21] Littell R.C., Pendergast J., Natarajan R. (2000). Modelling Covariance Structure in the Analysis of Repeated Measures Data. Stat. Med..

[22] Mello R., de Resende F.D., de Queiroz A.C., De M.H., De Oliveira A.S., Siqueira G.R. (2009). Bio-Economicity of the Finishing Phase on Feedlot of Crossbred Young Bulls Slaughtered at Different Body Weights. Rev. Bras. Zootec..

[23] Pazdiora R.D., de Resende F.D., de Faria M.H., Siqueira R., Benedito G., Almeida D.S., Sampaio R.L., Pacheco S., Sergio M., Prietto R. (2013). Animal Performance and Carcass Characteristics of Nellore Young Bulls Fed Coated or Uncoated Urea Slaughtered at Different Weights. Rev. Bras. Zootec..

[24] Rezende P.L.d.P., Restle J., Bilego U.O., Fernandes J.J.d.R., Missio R.L., Guimarães T.P. (2019). Carcass Characteristics of Feedlot-Finished Nellore Heifers Slaughtered at Different Weights. Acta Sci.—Anim. Sci..

[25] Silvestre A.M., Millen D.D. (2021). The 2019 Brazilian Survey on Nutritional Practices Provided by Feedlot Cattle Consulting Nutritionists. Rev. Bras. Zootec..

[26] Ferrari A.C., Berça A.S., Silva M.L.C., Leite R.G., Dallantonia E.E., Romanzini E.P., Barbero R.P., da Silva Cardoso A., Lage J.F., Tedeschi L.O. (2022). Effects of Supplement Type during the Pre-Finishing Growth Phase on Subsequent Performance of Nellore Bulls Finished in Confinement or on Tropical Pasture. Appl. Anim. Sci..

[27] Mota V.A.C., Fernandes R.M., Prados L.F., Alves Neto J.A., Berti G.F., Resende F.D., Siqueira G.R. (2020). Relationship between Gain Rate during the Growing Phase and Forage Allowance in the Finishing Phase in Nellore Cattle. Trop. Anim. Health Prod..

[28] Roth M.T.P., Resende F.D., Oliveira I., Fernandes R.M., Custódio L., Siqueira G.R. (2017). Does Supplementation during Previous Phase Influence Performance during the Growing and Finishing Phase in Nellore Cattle?. Livest. Sci..

[29] Simioni T.A., Torrecilhas J.A., Messana J.D., Granja-salcedo Y.T., Vito E.S., Lima A.R.C., Sanchez J.M.D., Reis R.A., Berchielli T.T. (2021). Influence of Growing-Phase Supplementation Strategies on Intake and Performance of Different Beef Cattle Genotypes in Finishing Phase on Pasture or Feedlot. Livest. Sci..

[30] Lancaster P.A., Krehbiel C.R., Horn G.W. (2014). A Meta-Analysis of Effects of Nutrition and Management during the Stocker and Backgrounding Phase on Subsequent Finishing Performance and Carcass Characteristics. Prof. Anim. Sci..

[31] Silva L.H.P., Paulino P.V.R., Assis G.J.F., Assis D.E.F., Estrada M.M., Silva M.C., Silva J.C., Martins T.S., Valadares Filho S.C., Paulino M.F. (2017). Effect of Post-Weaning Growth Rate on Carcass Traits and Meat Quality of Nellore Cattle. Meat Sci..

[32] Costa e Silva L.F., Valadares Filho S.C., Detmann E., Rotta P.P., Zanetti D., Villadiego F.A.C., Pellizzoni S.G., Pereira R.M.G. (2013). Performance, Growth, and Maturity of Nellore Bulls. Trop. Anim. Health Prod..

[33] Arboitte M.Z., Restle J., Filho D.C.A., Brondani I.L., Da Silva J.H.S., Nörnberg J.L., Kuss F. (2004). Desempenho Em Confinamento de Novilhos 5/8 Nelore-3/8 Charolês Abatidos Em Diferentes Estádios de Desenvolvimento. Rev. Bras. Zootec..

[34] Costa E.C., Restle J., Vaz F.N., Filho D.C.A., Bernardes R.A.L.C., Kuss F. (2002). Características Da Carcaça de Novilhos Red Angus Superprecoces Abatidos Com Diferentes Pesos. Rev. Bras. Zootec..

[35] Silva L.H.P., Paulino P.V.R., Benedeti P.D.B., Estrada M.M., Alves L.C., Assis D.E.F., Assis G.J.F., Leonel F.P., Valadares Filho S.C., Paulino M.F. (2020). Post-Weaning Growth Rate Effects on Body Composition of Nellore Bulls. Anim. Prod. Sci..

[36] Mesquita E.E., Castagnara D.D., De Oliveira N.T.E., Figueiredo A.C., Da Costa Oliveira A. (2016). Growth Performance and Carcass Characteristics of Nelore Angus and Nelore Angus Guzera Crossbreed Cows Fed with Supplemented Pasture during the Yearling and Feedlot Stages. Semin. Agrar..

[37] Pinto A.C.J., Millen D.D. (2018). Nutritional Recommendations and Management Practices Adopted By Feedlot Cattle Nutritionists : The 2016 Brazilian. Can. J. Anim. Sci..

[38] Williams C.B., Jenkins T.G. (2003). A Dynamic Model of Metabolizable Energy Utilization in Growing and Mature Cattle. I. Metabolizable Energy Utilization for Maintenance and Support Metabolism. J. Anim. Sci..

[39] May S.G., Dolezal H.G., Gill D.R., Ray F.K., Buchsnsn D.S. (1992). Effects of Days Fed, Carcass Grade Traits, and Subcutaneous Fat Removal on Postmortem Muscle Characteristics and Beef Palatability. J. Anim. Sci..

[40] Pflanzer S.B., Felício P.E. (2011). De Moisture and Fat Content, Marbling Level and Color of Boneless Rib Cut from Nellore Steers Varying in Maturity and Fatness. Meat Sci..

[41] De Oliveira I.M., Paulino P.V.R., Marcondes M.I., Valadares Filho S.D.C., Detmann E., Cavali J., Duarte M.D.S., Mezzomo R. (2011). Pattern of Tissue Deposition, Gain and Body Composition of Nellore, F 1 Simmental × Nellore and F 1 Angus × Nellore Steers Fed at Maintenance or *Ad Libitum* with Two Levels of Concentrate in the Diet. Rev. Bras. Zootec..

[42] Mueller L.F., Balieiro J.C.C., Ferrinho A.M., Martins T.d.S., Corte R.R.P.d.S., de Amorim T.R., Furlan J.d.J.M., Baldi F., Pereira A.S.C. (2019). Gender Status Effect on Carcass and Meat Quality Traits of Feedlot Angus × Nellore Cattle. Anim. Sci..

[43] Luchiari Filho A. (2000). Pecuária Da Carne Bovina.

[44] Pascoal L.L., Lobato J.F.P., Restle J., Vaz F.N., Vaz R.Z., de Menezes L.F.G. (2010). Beef Cuts Yield of Steer Carcasses Graded According to Conformation and Weight. Rev. Bras. Zootec..

[45] Missio R.L., Brondani I.L., Celestino D., Filho A., Restle J., Arboitte M.Z., Segabinazzi L.R. (2010). Características Da Carcaça e Da Carne de Tourinhos Terminados Em Confinamento, Recebendo Diferentes Níveis de Concentrado Na Dieta. Rev. Bras. Zootec..

